# Licorice extract inhibits growth of non-small cell lung cancer by down-regulating CDK4-Cyclin D1 complex and increasing CD8^+^ T cell infiltration

**DOI:** 10.1186/s12935-021-02223-0

**Published:** 2021-10-12

**Authors:** Jinglin Zhu, Ruifei Huang, Ruijie Yang, Yue Xiao, Jiangna Yan, Chunli Zheng, Wei Xiao, Chao Huang, Yonghua Wang

**Affiliations:** 1 Key Laboratory of Resource Biology and Biotechnology in Western China, Ministry of Education, School of Life Sciences, Northwest University, Xi’an, China; 2 State Key Laboratory of New-Tech for Chinese Medicine Pharmaceutical Process, Jiangsu Kanion Parmaceutical, Co., Ltd, Lianyungang, China; 3 Lab of Systems Pharmacology, Center of Bioinformatics, College of Life Science, Northwest A&F University, Yangling, 712100 China

**Keywords:** Tumor microenvironment, NSCLC, Licorice, Systems pharmacology strategy

## Abstract

**Background:**

Targeting tumor microenvironment (TME) may provide therapeutic activity and selectivity in treating cancers. Therefore, an improved understanding of the mechanism by which drug targeting TME would enable more informed and effective treatment measures. *Glycyrrhiza uralensis Fisch* (GUF, licorice), a widely used herb medicine, has shown promising immunomodulatory activity and anti-tumor activity. However, the molecular mechanism of this biological activity has not been fully elaborated.

**Methods:**

Here, potential active compounds and specific targets of licorice that trigger the antitumor immunity were predicted with a systems pharmacology strategy. Flow cytometry technique was used to detect cell cycle profile and CD8^+^ T cell infiltration of licorice treatment. And anti-tumor activity of licorice was evaluated in the C57BL/6 mice.

**Results:**

We reported the G0/G1 growth phase cycle arrest of tumor cells induced by licorice is related to the down-regulation of CDK4-Cyclin D1 complex, which subsequently led to an increased protein abundance of PD-L1. Further, in vivo studies demonstrated that mitigating the outgrowth of NSCLC tumor induced by licorice was reliant on increased antigen presentation and improved CD8^+^ T cell infiltration.

**Conclusions:**

Briefly, our findings improved the understanding of the anti-tumor effects of licorice with the systems pharmacology strategy, thereby promoting the development of natural products in prevention or treatment of cancers.

**Supplementary Information:**

The online version contains supplementary material available at 10.1186/s12935-021-02223-0.

## Introduction

Lung cancer is the most prevalent diagnosed cancer worldwide and a major contributor of cancer mortality. And non-small cell lung cancer (NSCLC) accounts for approximately 85% of the diagnosed lung cancers [1–3]. In recent years, immunotherapy targeting T cells has increasingly shown its potentiality in the treatment of a wide variety of solid tumors, such as NSCLC [4–6]. Although encouraging, it is the fact that still only a small  fraction  of patients obtain long-term benefit, which is likely correlated with the complex network of the tumor microenvironment (TME) [7]. TME, a complex physical and biochemical system, plays a pivotal role in tumor initiation, progression, metastasis, and drug resistance [8]. It contains cells of the immune system, tumor cells, tumor vasculature and extracellular matrices (ECM) [9]. Among them, tumor cells could express inhibitory ligands that suppress the T-cell activity to evade immune destruction. Immune cells could produce some cytokines, growth factors, enzymes, and angiogenic mediators to promote the growth of tumor [10]. And ECM consists of biological barriers around the tumor tissue to hamper lymphocyte penetration. Therefore, better understanding of the interactions in the TME would increase the ratio of patients benefiting from cancer therapies.

Traditional herb medicines and herbal derived components are playing increasingly critical roles in prevention and treatment of cancers [11, 12]. Compared with conventional chemotherapy, they are low toxicity and pleiotropic actions, targeting the complex network of TME by modulating multiple cell-signaling pathways involved in immune. Thereby, natural products could be a great repository for the development of novel therapeutic approaches in cancer treatment. As a well-known herbal medicine used worldwide for centuries, to date, several reports have published the immunomodulatory activity of licorice on multiple cancers, including colon cancer, breast cancer, acute myeloid leukemia, gastric cancer, melanoma, and prostate cancer [13–16]. However, the molecular underpinnings of licorice exert its immunomodulatory potential have not been fully elaborated.

To address this question, we used a systems pharmacology strategy [17] to elaborate that how licorice exerts anti-tumor effects by regulating multiple immune-related signaling pathways and targets, influencing cell cycle progression, and mitigates the growth of NSCLC cancer. First, by screening the poly-pharmacology molecules of licorice, predicting the targets of active compounds, constructing the networks, and linking the targets to the immune phenotype in lung cancer patients, we observed that the active ingredients of licorice targeted a great variety of tumor-related signaling pathways, including cell cycle, inflammation, and migration. Then, we used in vitro and in vivo experiments to reveal the anti-tumor effects of licorice. On the one hand, we found that licorice down-regulates CDK4-Cyclin D1 complex, resulting in G0/G1 phase arrest and increased PD-L1 levels in lung cancer cells. On the other hand, we also found that licorice increased antigen presentation and infiltration of CD8^+^ T cell, significantly decreased tumor volume of mouse models of NSCLC in vivo. Taken together, our studies indicate that the systems pharmacology strategy greatly uncovered the action mechanism of poly-pharmacology molecules of licorice, contributing the use of natural products for further anti-cancer drug development.

## Results

### Systems pharmacology uncovers that licorice targets cell cycle progression and immune process

**Fig. 1:**
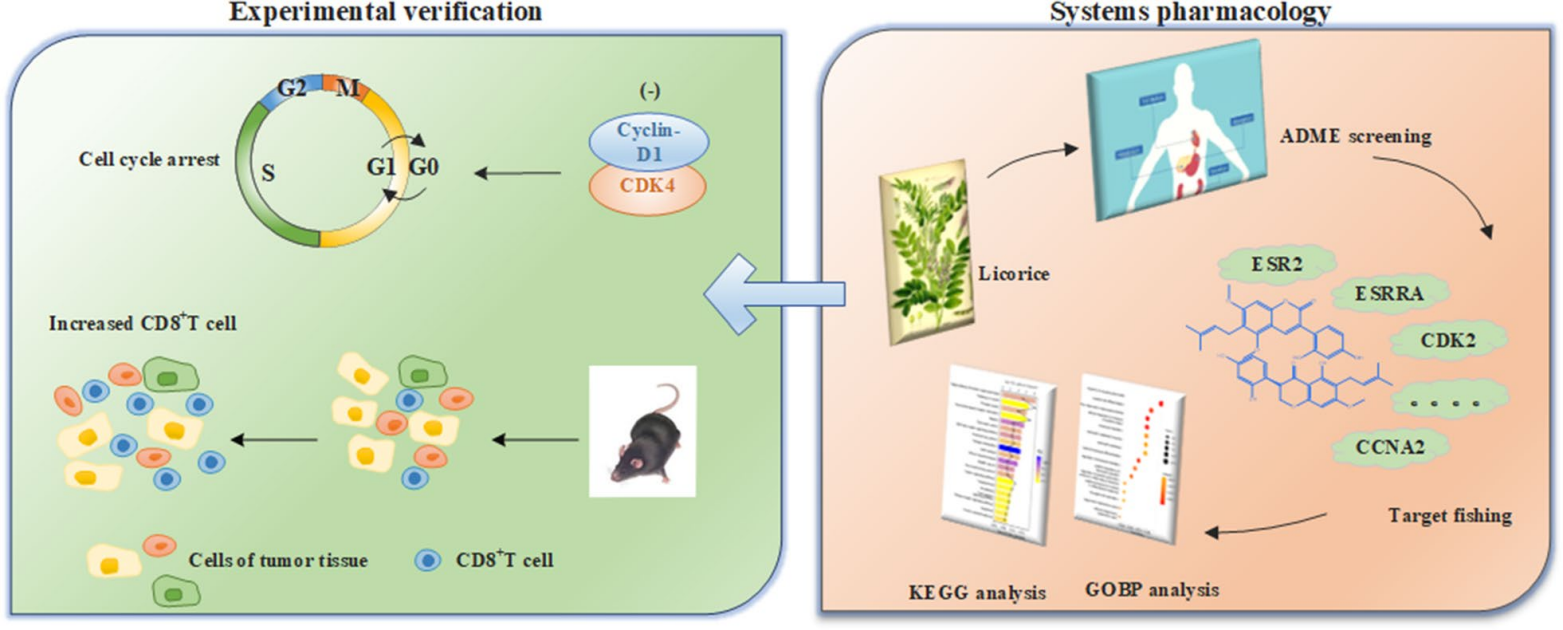
Workflow of systems pharmacology analysis to uncover mechanism of licorice

As a comprehensive system, the systems pharmacology approach was used to investigate the complex molecular mechanisms of licorice as a treatment for NSCLC in this study (as shown in Fig. 1).

Altogether, 280 ingredients were identified in licorice with the searching literatures and using TCMSP and BATMAN-TCM, and a total of 23 active ingredients (shown in Table 1) with higher druggability were screened out by in silico ADME (absorption, distribution, metabolism, and excretion) system, with the criteria of oral bioavailability (OB) ≥ 50% and drug-likeness (DL) ≥ 0.40. Take Liquiritin as an example, it was predicted with OB = 65.69% and DL = 0.74, and inhibited H1975 cells growth significantly after 48 h treatments (Fig. 1a). The inhibitory effect by Liquiritin was also presented in the growth of colon carcinoma cell lines [18] and human cervical cancer cell lines [19]. These results demonstrated the validity of the raised criteria on screening potential druggability compounds for anti-tumor treatment.

**Table 1:** Chemical information and pharmacokinetics parameters of the 23 active compounds of licorice

MOL-ID	Compounds	Structure	Categories	OB	DL	Degree
MOL005008	Glycyrrhiza flavonol A		Flavonoids	41.28	0.60	30
MOL001484	Inermine		Flavonoids	75.18	0.54	35
MOL000211	Mairin		Saponins	55.38	0.78	16
MOL002311	Glycyrol		Coumestans	90.78	0.67	14
MOL004808	Glyasperin B		Others	65.22	0.44	31
MOL004810	Glyasperin F		Others	75.84	0.54	33
MOL004820	Kanzonols W		Flavonoids	50.48	0.52	38
MOL004855	Licoricone		Flavonoids	63.58	0.47	23
MOL004863	3-(3,4-Dihydroxyphenyl)-5,7-dihydroxy-8-(3-methylbut-2-enyl)chromon		Others	66.37	0.41	26
MOL004879	Glycyrin		Coumarins	52.61	0.47	22
MOL004885	Licoisoflavanone		Flavonoids	52.47	0.54	31
MOL004891	Shinpterocarpin		Flavonoids	80.3	0.73	44
MOL004903	Liquiritin		Flavonoids	65.69	0.74	21
MOL004904	Licopyranocoumarin		Flavonoids	80.36	0.65	25
MOL004908	Glabridin		Flavonoids	53.25	0.47	39
MOL004912	Glabrone		Flavonoids	52.51	0.5	38
MOL004914	1,3-Dihydroxy-8,9-dimethoxy-6-benzofurano[3,2-c]chromenone		Others	62.9	0.53	20
MOL004959	1-Methoxyphaseollidin		Flavonoids	69.98	0.64	35
MOL005001	Gancaonin H		Others	50.1	0.78	30
MOL005003	Licoagrocarpin		Flavonoids	58.81	0.58	37
MOL005007	Glyasperins M		Flavonoids	72.67	0.59	39
MOL005012	Licoagroisoflavone		Flavonoids	57.28	0.49	36
MOL005017	Phaseol		Coumestans	78.77	0.58	21

OB: oral bioavailability; DL: drug-likeness

**Fig. 2:**
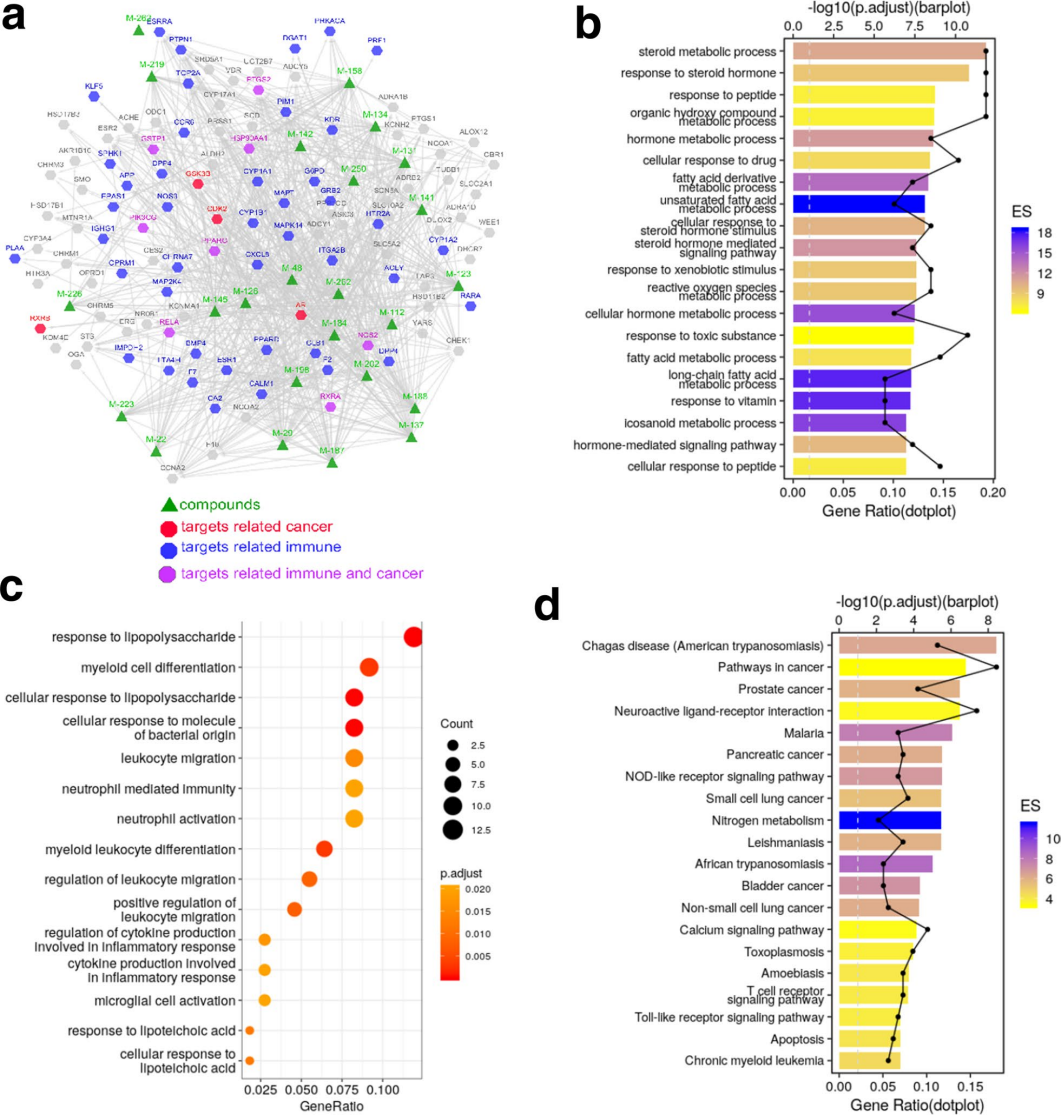
Systems pharmacology analysis of targets of licorice. **a** Construction of compound-target network, the triangle represents compounds, the octagon represents targets, the edge represents connection between compounds and targets. **b** GO enrichment analysis of potential targets of licorice, the y-axis represents the enriched GO terms, and the GeneRatio represents the number of targets located in this GO/the total number of targets located in the GO. **c** GO terms associated with immune process were shown, and the size of the circle represents the count. **d** KEGG analysis of targets of licorice, the color represents the enrichment significance, the y-axis represents pathways, and the GeneRatio represents the number of targets located in this KEGG pathways/the total number of targets located in the KEGG pathways

Then, predicted by the weighted ensemble similarity method (WES) [20] and systematic drug targeting tool (SysDT) [21], we found that these 23 ingredients in licorice were investigated interacted with 109 targets (shown in Table 2 and Additional file 1: Table S1). And we constructed the compound-target (C-T) network graph to greatly illustrate the relationships between compounds and targets. In terms of the targets interacted with licorice, we observed that most of which were related to cell cycle, immune, inflammation, cancer and neoplasm metastasis with higher scores,  specifically,  such as CDK2, ESR1, PPARG, ESRRA, PRKACA, CXCL8, PLAA, RXRB, MAPK14 and so on (shown in Fig. 2a).

**Table 2:** The information of licorice’s targets

UniProt-ID	Protein names	Gene names	Degree	Species
P0DP23	Calmodulin-1	CALM1	19	*Homo sapiens*
P35368	Alpha-1B adrenergic receptor	ADRA1B	5	homo sapiens
P00918	Carbonic anhydrase 2	CA2	17	*Homo sapiens*
P18031	Tyrosine-protein phosphatase non-receptor type 1	PTPN1	17	*Homo sapiens*
P46098	5-hydroxytryptamine receptor 3A	HTR3A	1	*Homo sapiens*
P20309	Muscarinic acetylcholine receptor M3	CHRM3	3	*Homo sapiens*
P23219	Prostaglandin G/H synthase 1	PTGS1	8	*Homo sapiens*
Q14524	Sodium channel protein type 5 subunit alpha	SCN5A	11	*Homo sapiens*
P07477	Trypsin-1	PRSS1	18	*Homo sapiens*
P17612	cAMP-dependent protein kinase catalytic subunit alpha	PRKACA	6	*Homo sapiens*
O14757	Serine/threonine-protein kinase	CHEK1	18	*Homo sapiens*
P11309	Serine/threonine-protein kinase pim-1	PIM1	20	*Homo sapiens*
P35354	Prostaglandin G/H synthase 2	PTGS2	20	*Homo sapiens*
P27487	Dipeptidyl peptidase 4	DPP4	13	*Homo sapiens*
Q16539	Mitogen-activated protein kinase 14	MAPK14	13	*Homo sapiens*
P48736	Phosphatidylinositol 4,5-bisphosphate 3-kinase catalytic subunit gamma isoform	PIK3CG	3	*Homo sapiens*
P21730	C5a anaphylatoxin chemotactic receptor 1	AR	22	*Homo sapiens*
P49841	Glycogen synthase kinase-3 beta	GSK3B	17	*Homo sapiens*
P24941	Cyclin-dependent kinase 2	CDK2	17	*Homo sapiens*
Q92731	Estrogen receptor beta	ESR2	16	*Homo sapiens*
P07900	Heat shock protein HSP 90-alpha	HSP90AA1	12	*Homo sapiens*
P20248	Cyclin-A2	CCNA2	20	*Homo sapiens*
B2RXH2	Lysine-specific demethylase 4E	KDM4E	1	*Homo sapiens*
O00767	Stearoyl-CoA desaturase	SCD	10	*Homo sapiens*
O95622	Adenylate cyclase type 5	ADCY5	7	*Homo sapiens*
P08842	Steryl-sulfatase	STS	13	*Homo sapiens*
P11474	Steroid hormone receptor ERR1	ESRRA	12	*Homo sapiens*
P12644	Bone morphogenetic protein 4	BMP4	1	*Homo sapiens*
P16152	Carbonyl reductase [NADPH] 1	CBR1	7	*Homo sapiens*
P28223	5-hydroxytryptamine receptor 2A	HTR2A	18	*Homo sapiens*
P51843	Nuclear receptor subfamily 0 group B member 1	NR0B1	7	*Homo sapiens*
Q99814	Endothelial PAS domain-containing protein 1	EPAS1	3	*Homo sapiens*
Q9Y263	Phospholipase A-2-activating protein	PLAA	3	*Homo sapiens*
O60218	Aldo–keto reductase family 1 member B10	AKR1B10	1	*Homo sapiens*
P05093	Steroid 17-alpha-hydroxylase/17,20 lyase	CYP17A1	1	*Homo sapiens*
P10276	Retinoic acid receptor alpha	RARA	1	*Homo sapiens*
P11413	Glucose-6-phosphate 1-dehydrogenase	G6PD	1	*Homo sapiens*
P11473	Vitamin D3 receptor	VDR	1	*Homo sapiens*
P16662	UDP-glucuronosyltransferase 2B7	UGT2B7	1	*Homo sapiens*
P18405	3-oxo-5-alpha-steroid 4-dehydrogenase 1	SRD5A1	1	*Homo sapiens*
P19793	Retinoic acid receptor RXR-alpha	RXRA	7	*Homo sapiens*
P36873	Serine/threonine-protein phosphatase PP1-gamma catalytic subunit	PPP1CC	1	*Homo sapiens*
P80365	Corticosteroid 11-beta-dehydrogenase isozyme 2	HSD11B2	2	*Homo sapiens*
Q08828	Adenylate cyclase type 1	ADCY1	1	*Homo sapiens*
Q12908	Ileal sodium/bile acid cotransporter	SLC10A2	1	*Homo sapiens*
Q9NRD8	Dual oxidase 2	DUOX2	1	*Homo sapiens*
Q9UBM7	7-dehydrocholesterol reductase	DHCR7	1	*Homo sapiens*
P03372	Estrogen receptor	ESR1	13	*Homo sapiens*
P03420	Fusion glycoprotein F2	F2	18	*Homo sapiens*
P37231	Peroxisome proliferator-activated receptor gamma	PPARG	19	*Homo sapiens*
P30291	Wee1-like protein kinase	WEE1	3	*Homo sapiens*
P23141	Liver carboxylesterase 1	CES2	7	*Homo sapiens*
P05067	Amyloid-beta precursor protein	APP	7	*Homo sapiens*
P09960	Leukotriene A-4 hydrolase	LTA4H	10	*Homo sapiens*
P10636	Microtubule-associated protein tau	MAPT	9	*Homo sapiens*
Q04206	Transcription factor p65	RELA	6	*Homo sapiens*
P22303	Acetylcholinesterase	ACHE	11	*Homo sapiens*
Q15596	Nuclear receptor coactivator 2	NCOA2	10	*Homo sapiens*
P11388	DNA topoisomerase 2-alpha	TOP2A	11	*Homo sapiens*
P35968	Vascular endothelial growth factor receptor 2	KDR	8	*Homo sapiens*
P00742	Coagulation factor X	F10	16	*Homo sapiens*
P08709	Coagulation factor VII, EC 3.4.21.21	F7	7	*Homo sapiens*
P11926	Ornithine decarboxylase	ODC1	10	*Homo sapiens*
P14061	17-beta-hydroxysteroid dehydrogenase type 1	HSD17B1	5	*Homo sapiens*
P18054	Olyunsaturated fatty acid lipoxygenase ALOX12	ALOX12	7	*Homo sapiens*
Q9UHC3	Acid-sensing ion channel 3	ASIC3	12	*Homo sapiens*
P05091	Aldehyde dehydrogenase	ALDH2	4	*Homo sapiens*
P37058	Testosterone 17-beta-dehydrogenase 3	HSD17B3	3	*Homo sapiens*
Q13887	Krueppel-like factor 5	KLF5	2	*Homo sapiens*
Q15788	Nuclear receptor coactivator 1	NCOA1	6	*Homo sapiens*
Q12809	Potassium voltage-gated channel subfamily H member 2	KCNH2	5	*Homo sapiens*
Q9H4B7	Tubulin beta-1 chain	TUBB1	5	*Homo sapiens*
P12268	Inosine-5'-monophosphate dehydrogenase 2	IMPDH2	1	*Homo sapiens*
P11308	Transcriptional regulator ERG	ERG	1	*Homo sapiens*
P45985	Dual specificity mitogen-activated protein kinase kinase 4	MAP2K4	1	*Homo sapiens*
P25100	Alpha-1D adrenergic receptor	ADRA1D	2	*Homo sapiens*
P36544	Neuronal acetylcholine receptor subunit alpha-7	CHRNA7	1	*Homo sapiens*
P28702	Retinoic acid receptor RXR-beta	RXRB	2	*Homo sapiens*
P08912	Muscarinic acetylcholine receptor M5	CHRM5	1	*Homo sapiens*
P11229	Muscarinic acetylcholine receptor M1	CHRM1	2	*Homo sapiens*
P07550	Beta-2 adrenergic receptor	ADRB2	4	*Homo sapiens*
P35372	Mu-type opioid receptor	OPRM1	1	*Homo sapiens*
P41143	Delta-type opioid receptor	OPRD1	1	*Homo sapiens*
O60502	Protein *O*-GlcNAcase	OGA	1	homo sapiens
P08514	Integrin alpha-IIb	ITGA2B	1	*Homo sapiens*
P16278	Beta-galactosidase	GLB1	1	*Homo sapiens*
P28838	Cytosol aminopeptidase	LAP3	1	*Homo sapiens*
P31639	Sodium/glucose cotransporter 2	SLC5A2	1	*Homo sapiens*
P53396	ATP-citrate synthase	ACLY	1	*Homo sapiens*
P54577	Tyrosine–tRNA ligase, cytoplasmic	YARS	1	*Homo sapiens*
O75907	Diacylglycerol O-acyltransferase 1	DGAT1	3	*Homo sapiens*
P14222	Perforin-1	PRF1	1	*Homo sapiens*
P51684	C–C chemokine receptor type 6	CCR6	2	*Homo sapiens*
P05177	Cytochrome P450 1A2	CYP1A2	1	*Homo sapiens*
Q16678	Cytochrome P450 1B1	CYP1B1	1	*Homo sapiens*
Q92959	Solute carrier organic anion transporter family member 2A1	SLCO2A1	1	*Homo sapiens*
P29474	Nitric oxide synthase	NOS3	2	*Homo sapiens*
P08684	Cytochrome P450 3A4	CYP3A4	1	*Homo sapiens*
P09211	Glutathione S-transferase P	GSTP1	2	*Homo sapiens*
Q99835	Smoothened homolog	SMO	1	*Homo sapiens*
Q9NYA1	Sphingosine kinase 1	SPHK1	1	*Homo sapiens*
P48039	Melatonin receptor type 1A	MTNR1A	1	*Homo sapiens*
Q03181	Peroxisome proliferator-activated receptor delta	PPARD	1	*Homo sapiens*
P10145	Interleukin-8	CXCL8	1	*Homo sapiens*
P62993	Growth factor receptor-bound protein 2	GRB2	1	*Homo sapiens*
P01857	Immunoglobulin heavy constant gamma 1	IGHG1	2	*Homo sapiens*
P35228	Nitric oxide synthase	NOS2	20	*Homo sapiens*
P04798	Cytochrome P450 1A1	CYP1A1	4	*Homo sapiens*
Q12791	Calcium-activated potassium channel subunit alpha-1	KCNMA1	1	*Homo sapiens*

To analyze the biological processes in which the targets of the bioactive ingredients participated, we implemented Gene Ontology (GO) biological processes enrichment analysis. As shown in Fig. 2b, metabolic processes or pathways of steroid, hormone and fatty acid were enriched, such as “steroid metabolic process”, “hormone metabolic process” and “fatty acid derivative metabolic process”, which are the most prominent metabolic alterations in cancer [22], 23. The biological processes of “response to peptide”, “cellular response to peptide” were also enriched, which are important ways to stimulate the acquired immune system. Thus, enrichment analysis of targets demonstrated that licorice has the potential anti-tumor effects by regulating tumor cell viability and anti-tumor immunity [24, 25].

To further clarify the relationship between licorice targets and biological processes of immunity, we therefore screened out the immune-related GOBP terms (Fig. 2c) and found that terms of differentiation, activation and migration of innate immune cells, myeloid cell, leukocyte or neutrophil were top enriched. Several inflammation-associated processes were also presented including “ regulation of cytokine production involved in inflammatory response”, “response to lipopolysaccharide”, etc., which involve in the processes of tumor growth such as angiogenesis [26–28]. These biological processes showed that the targets of bioactivate compounds are closely related to cancer.

In the list of the TOP 20 pathways, 10 significantly enriched pathways involved in cancer and immune were found by the KEGG Pathway analysis (Fig. 2d). A cohort of pathways (7/10) directly related to cancer, for example, “Pathways in cancer”, “Prostate cancer”, “Pancreatic cancer”, “Small cell lung cancer”, “Bladder cancer”, “non-small cell lung cancer”, and “Chronic myeloid leukemia” were enriched. Besides, “NOD-like receptors signaling pathway”, “T cell receptor signaling pathway”, and “Toll-like receptor signaling pathway”, these immune related pathways (3/10) were also identified. “Apoptosis” and “Calcium signaling pathway” were also showed in the chart which were downstream process or signaling pathway in cancer development. All the data indicated the reliability of the potential effect of compounds of licorice on cancer treatment.

Therefore, the systems pharmacology analysis uncovers that licorice mainly targets cancer cell and immune progress to exert its anti-cancer effect, and paves the way for in-depth understanding of the multi-target molecular mechanism of licorice treating for NSCLC.

### Licorice induced tumor cells cycle arrest mainly by down-regulating Cyclin D1-CDK4

**Fig. 3:**
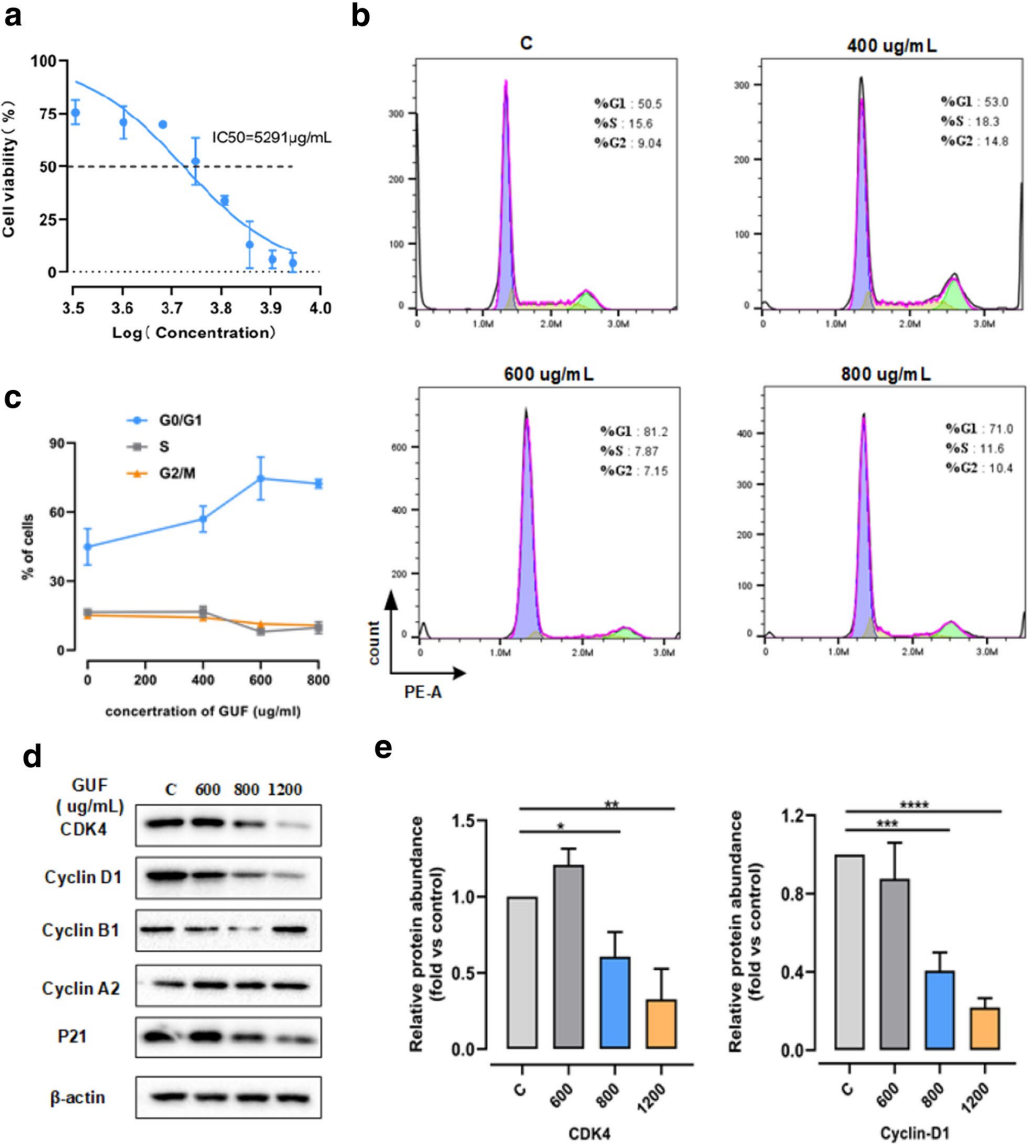
Licorice induces tumor cell G0/G1 phase arrested with the degradation of CDK4-Cyclin D1 complex. **a** H1975 cells were treated with different concentrations of licorice for 48 h, cell viability was determined using the CCK-8 assay. (mean ± SD, n = 6). **b** The cell-cycle profiles of H1975 cells incubated with 400, 600, 800 µg/ml GUF or vehicle control for 48 h were shown by using fluorescence-activated cell sorting (FACS). **c** Percentages of H1975 cells in **b** at different cell cycle states. **d** The protein expression in H1975 cells pretreated with 400,  600, 800 µg/ml GUF or vehicle control were measured by western blot anaylsis , versus β-actin as a loading control. **e** Relative protein abundance of CDK4 and Cyclin D1 of **d**. *p < 0.05, **p < 0.01, ***p < 0.001, ****p < 0.0001

To further study the anti-cancer effect of licorice on NSCLC, we firstly tested the effects of licorice on the growth of tumor cells. According to the CCK8 assay results shown in Fig. 3a, we could recognize that licorice induced a concentration-dependent inhibition of H1975 cell proliferation. Treating licorice 2 days with concentrations of 3200, 5600 and 7200 μg/mL, we found that compared to the control group, the H1975 cell growth decreased by 25, 48 and 87%, respectively. Moreover, the IC50 value on it is  ~ 5400 μg/mL.

Next, given the analysis of systems pharmacology for licorice, and a number of studies have shown that the negative effects of licorice or its relatives on cell cycle progression [15, 16, 29, 30], we reasoned that licorice might influenced cell cycle to exert the anti-tumor effect on NSCLC to some extent. To test the hypothesis, we treated H1975 cells with different concentrations of licorice followed by flow cytometry analysis of cell cycle profile. Strikingly, H1975 cells subjected to licorice led to a significant increase in the number of cells arrested at G0/G1 growth phase, in a dose-dependent manner, compared with vehicle control containing media (shown in Fig. 3b and c). At the same time, the number of cells at both S growth phase and G2/M growth phase slightly decreased (Fig. 3c). This findings consistent with previous study that licorice induced G1 cell cycle arrest in MCF-7 human breast cancer cells [16].

It has been known that cyclin-dependent kinase (CDK)/cyclin complexes, such as CDK2/Cyclin E, CDK4, CDK6/Cyclin D1, and P21 play crucial roles in cell cycle progression [31]. Therefore, to elucidate the underlying molecular mechanism with which licorice induced cell cycle arrest at G0/G1 growth phase, immunoblot analysis were performed to evaluate cell cycle-related protein abundance in vitro experiment. Notably, we found that the levels of CDK4, cyclin D1 were reduced with concentration dependent, while the expressions of Cyclin B1 and Cyclin A2 were  relatively maintained at the level of the control group following licorice treatment (Fig. 3d and e). Interestingly, the expression of p21, a CDK inhibitor, was slightly decreased in response to licorice exposure vs control group (shown in Fig. 3d).

In addition, previous works uncovered that cyclin D1 degradation occurs mainly at the G1/S phase boundary [31, 32]. Collectively, these results indicated that licorice is likely to induce tumor cells arrested at G0/G1 growth phase by down-regulating CDK4-Cyclin D1 complex.

### Licorice positively regulates PD-L1 protein abundance

**Fig. 4:**
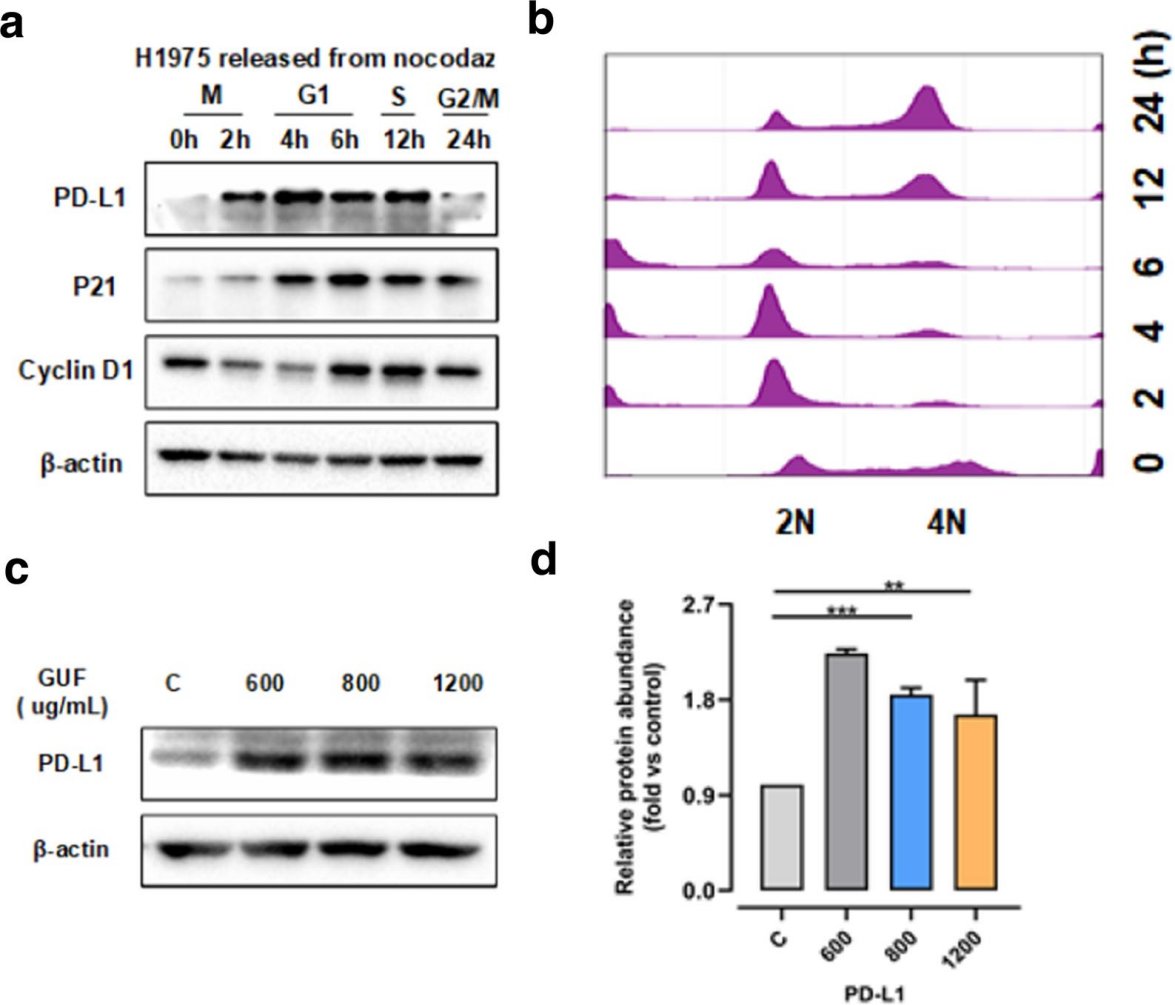
Licorice induces increase of expression level of PD-L1. **a** Western blot results of whole cell lysates derived from H1975 cells synchronized in M phase by nocodazole treatment prior to releasing back into the cell cycle for the indicated times. **b** The cell-cycle profiles in **a** were monitored by FACS. **c** The protein expression in H1975 cells pretreated with 400, 600, and 800 µg/ml GUF or vehicle control, was measured by  western blot, versus β-actin as a loading control. **d** Relative protein abundance of PD-L1 of **c**. **p < 0.01, ***p < 0.001

It has been shown that PD-L1 expression can be modulated at both transcriptional and post-translational levels, however, it is not yet clear whether PD-L1 expression is regulated under physiological conditions for example during cell cycle progression [33–36]. In this setting, to further understand the connection between PD-L1 and cell cycle, we used cell synchronization by nocodazole arrest and western blot analysis to explore variation of PD-L1 during cell cycle. As shown in Fig. 4a and b, we found that PD-L1 protein expression increased in M/early G1 phases, followed by a great decrease in late G1/S phases.

As our results showed that licorice down-regulates CDK4-Cyclin D1expression to arrest cell cycle progression, we probed whether licorice participated in variation of PD-L1. To do this, we treated H1975 cells with different concentration of licorice extract, followed by western blot analysis. Strikingly, licorice administration result in a significant increase in the expression of PD-L1 protein (Fig. 4c and d), in a dose-dependent manner. Similar to H1975 cells, we also found that licorice down-regulates CDK4-Cyclin D1 complex and leads to increased protein abundance of PD-L1 in A549 cells subjected to different concentrations of licorice (Fig. 3). Furthermore, recent finding had shown that CDK4-Cyclin D 1 kinase destabilized PD-L1, inhibition of CDK4/6 in vivo increased PD-L1 protein levels [37]. Together, these findings indicated that increased levels of PD-L1 expression by licorice correlated with down-regulation of CDK4-Cyclin D1 expression.

### Licorice induces tumor regression by affecting CDK4-Cyclin D1

**Fig. 5:**
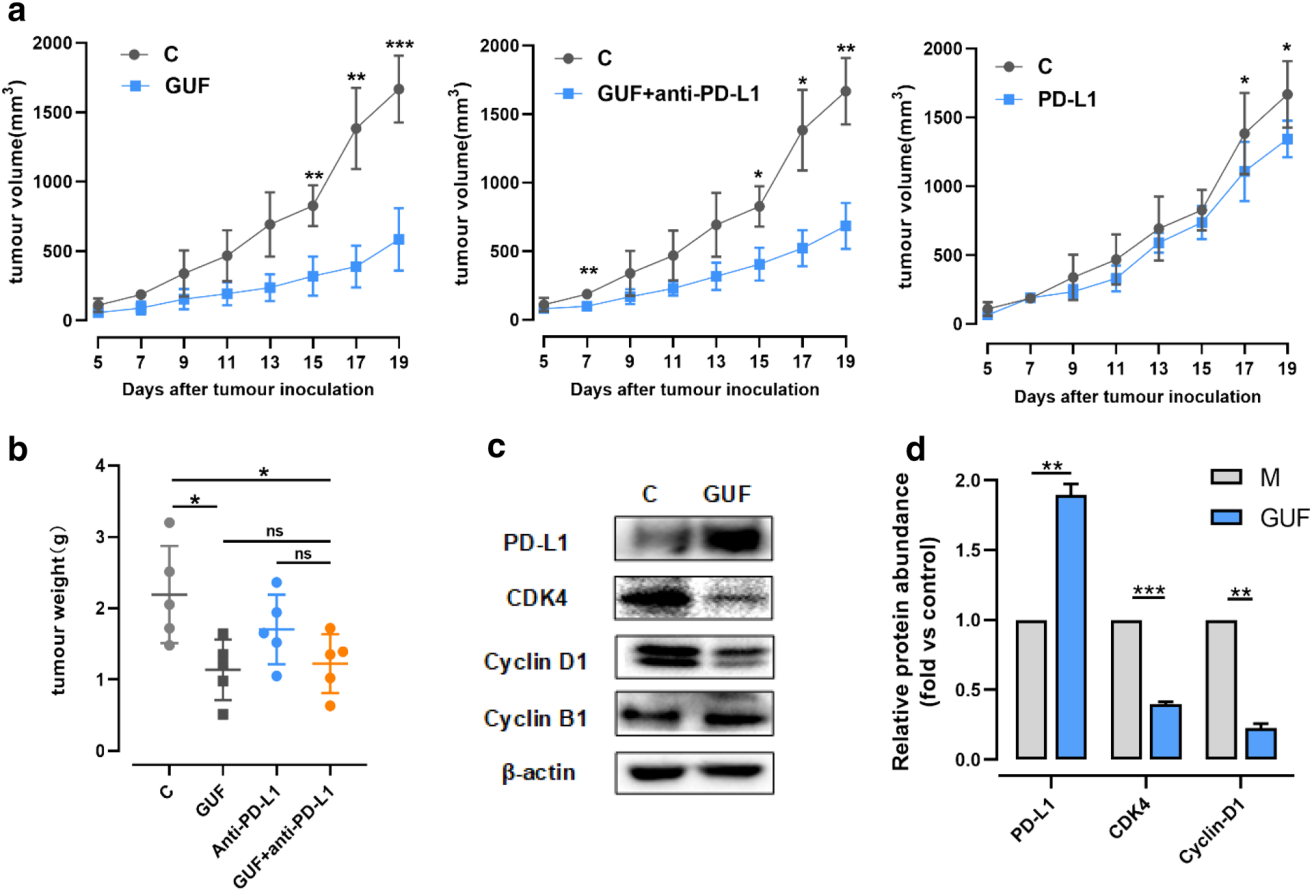
Licorice inhibits the growth of tumor volume depending on the CDK4-Cyclin D1 axis. **a** C57BL/6 mice were injected with 5×10^5^ LLC cells. 24 h later, 200 mg/kg GUF or vehicle were administered once daily from day 2 and/or 200 μg/mouse anti-PD-L1 (i.p.) on day 4, 7, 10 (n = 5 per group). The tumor growth curve is shown, with tumor sizes presented as mean ± SD. **b** Primary tumor mass of mice is shown, presented as mean ± SD. **c** Protein expression in tumors from GUF group and control group was measured by western blot, versus β-actin as a loading control. **d** Relative protein abundance of PD-L1, CDK4, Cyclin D1, Cyclin B1 of **c**, *p < 0.05, **p < 0.01, ***p < 0.001, n.s.: no significant

Based on previous studies that various natural compounds in licorice possess effective antitumor activity [14, 16, 38, 39], we wanted to know whether licorice can function in vivo to suppress tumor progression for NSCLC. To do so, we utilized C57/BL6 female mice bearing LLC tumor to assess the anti-tumor impact of licorice. And the size-matched tumor-bearing mice were divided into 4 groups randomly and received the administrations (as depicted in Fig. 5a).

By day 20 of treatment, as expected, all control mice encountered humane endpoints. Then mice from each group were killed and dissected tumor, mouse serum was taken out and stored for subsequent experiments.

It is critical to note that licorice treatment result in a 64.9% tumor volume regression, and we found that there was slightly inhibitory effect on tumor volume of mice treated with anti-PD-L1 antibody alone vs control mice. Interestingly, we also observed a 54.7% tumor volume reduction in licorice + anti-PD-L1 mice compared with control mice over time (Fig. 5a).

Consistent with the observed reduction in tumor volume, treatment of licorice led to a significant induction of tumor weight, this also occurred in licorice + anti-PD-L1 group compared with untreated group. However, slight reduction of tumor weight was observed in anti-PD-L1 alone group (Fig. 5b). No significant loss of mice body weight was displayed among all the groups throughout the period of the experiment (Additional file 1: Fig. S4a).

Having pinpointed the critical role for licorice in affecting Cyclin D1-CDK4 expression in vitro, we next examined whether licorice had similar influence in vivo. Therefore, we assayed cell cycle-related protein for tumor tissue using the  western blot assay. Consistent with earlier observation in vitro (Fig. 3d), licorice treatment markedly reduced the abundance of CDK4 and Cyclin D1, and led to a dramatic PD-L1 accumulation compared with control group significantly (Fig. 5c and d).

Therefore, these results coherently indicated that licorice might mainly function through down-regulating CDK4-Cyclin D1 to stabilize PD-L1 and subsequently suppress tumor progression.

### Licorice increases antigen presentation and infiltration of CD8^+^ T cell

**Fig. 6:**
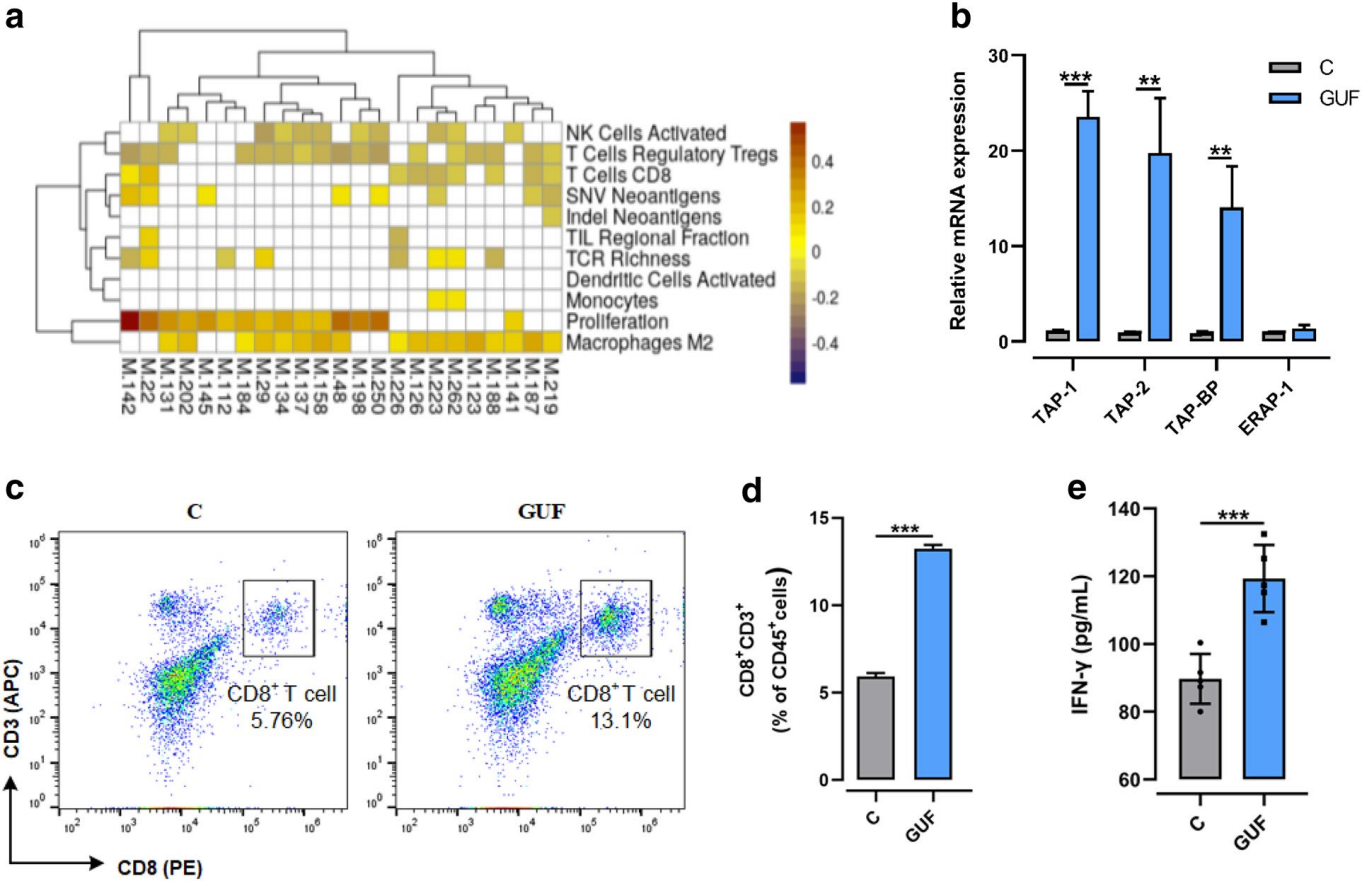
Licorice increases antigen presentation and infiltration of CD8^+^ T cells in vivo. **a** Heatmap of Pearson’s correlation coefficients (PCCs) between gene expression level of targets of licorice and immune phenotypes in TCGA LUAD dataset. For the targets of each active compound, we used the GSVA method to evaluate the overall expression level of targets based on the gene expression profiles of LUAD patients. The y-axis represents the compounds of licorice. **b** Relative Quantitative real-time PCR (q RT-PCR) analyses of relative mRNA levels of antigen presentation gene from licorice-treated tumors or vehicle. The experiments were repeated three times. Data was analyzed using ANOVA test. **c** Freshly isolated lymphocytes of tumor tissue samples from the GUF-treated and control groups were stained with anti-CD45 (PE-Cy7), anti-CD3 (APC), and anti-CD8 (PE) antibodies and infiltration of CD8^+^ T cells examined by FACS. Representative flow-cytometry plots were shown. **d** Ratio of infiltration of CD8^+^ T cells in mice tumor samples from the GUF-treated group versus control group. **e** Bar graph of IFN-γ levels based on ELISA in mice tumor samples from the GUF-treated group and control group were shown, (n = 5). **p < 0.01, ***p < 0.001

Furthermore, we showed that targets of licorice active compounds correlated with CD8^+^ T-cell infiltration in TCGA LUAD patients . And the immune phenotypes of TCGA LUAD patients were evaluated by Thorsson et al. [40] , (Fig. 6a, Additional file 1: Figs. S4b, c). Then intratumoral CD8^+^ T-cell infiltration in tumor tissue lysates were measured by flow cytometry analysis. To this end, a flow cytometry staining protocol was established to identify CD8^+^ T cell populations in tumors. We manually analyzed the flow cytometry data using a common rational gating strategy included three gate events as follows in the work: total number of all live-gated events, immune compartment gate events, and CD8^+^ T gate events (Additional file 1: Figure S4d). Importantly, CD8^+^ T cell infiltration of licorice-treated mice we detected increased by 6% of that in untreated mice (Fig. 6c and d). To further support of the physiological role for licorice in promoting CD8^+^ T cell infiltration, we used the mice serum to perform ELISA-based assays and found a remarkable increase of IFN-γ in licorice-treated mice (Fig. 6e). These results were in line with a previous study that CDK4/6 inhibitors induce breast cancer cell cytostasis and enhance their capacity to present antigen and stimulate cytotoxic T cells [41].

Next, to gain insights into the physiological role of licorice in modulating tumor regression at a gene level, RT-qPCR analysis was performed. Specifically, we sought to determine relative mRNA levels of antigen presentation genes by RT-qPCR analysis, and observed that transporter–MHC interactions (Tap-bp) had at least a 15 × fold increase in licorice-treated tumor tissues compared to control tumor samples. And peptide transporters (Tap1 and Tap2) were also markedly up-regulated in licorice-treated tumors, although directing peptide cleavage (Erap1) hardly change to some extent (Fig. 6b).

Altogether, these studies indicated that licorice increased expression of antigen presentation genes and promoted CD8^+^ T cell infiltration of  tumor tissue.

## Discussion

Natural products were shown broadly to interfere growth signals by multi-specific actions [42], which may open an opportunity to treat NSCLC effectively. In a panel of human cancers, licorice has been uncovered to provide growth-limiting activities [16, 38, 43]. Although changes in the cell-cycle have been noted under licorice treatment settings [29, 30], dissecting mechanism of the biological activity of licorice remains a challenge. Here, the critical findings of our study, summarized in Figs. 5c and 2d, include the discovery that licorice limits lung cancer growth mainly related with down-regulating CDK4-Cyclin D1 complex and enhancing intra-tumoral CD8^+^ T cell infiltration. Our detailed investigation shows that licorice induces G1 cell-cycle arrest in lung cancer cells by inhibiting CDK4-Cyclin D1 complex, which in turn increases antigen presentation and results in intra-tumoral CD8^+^ T cell infiltration but increase PD-L1 levels. These findings convincingly argue for a potential treatment option of licorice in the prevention and treatment of NSCLC.

Beginning with systems pharmacology analysis, flow cytometry analysis of cell cycle profile and western blot, we observed that licorice treatment led to G1 cell-cycle arrest and inhibit the expression of CDK4-Cyclin D1 complex in H1975 cells. This biological activity was further validated in licorice-treated tumor. It is well known that CDK4-Cyclin D1 complex were required for progression of cells cycle through the G0/G1 phase [44–46], which would suggest that G1 cell-cycle arrest is largely associated with decreased levels of CDK4-Cyclin D1 after licorice treatment. The tumor regression caused by down-regulation of CDK4-Cyclin D1 complex has been demonstrated in CDK4/CDK6 inhibitor studies. As a kind of CDK4/6 inhibitors, abemaciclib caused regression of bulky tumors in mouse models of mammary carcinoma [41]. Furthermore, many human cancers harbor genomic or transcriptional aberrations that could activate CDK4/6 [47–49]. Therefore, our findings revealed that licorice inhibit the expression of CDK4-Cyclin D1 complex would be critically important for prevention and treatment of lung cancers.

Moreover, CDK4-Cyclin D was found negatively regulates PD-L1 protein stability in several tumor cell lines [37, 50]. And previous studies revealed that response to PD-1/PD-L1 blockade might correlate with PD-L1 expression levels in tumor cells [51–53]. Notably, we discovered that licorice treatment induced increased expression of PD-L1 levels both in vitro and in vivo. These studies, together with our findings, shed light on a viable option for the management of NSCLC, with or without other treatments in conjunction, to enhance the efficiency of cancer immunotherapies.

The functional impairment of T cell-mediated immunity in the TME is a defining feature sharing by many cancers, and CD8^+^ T cells became the central focus of new cancer therapeutics [54, 55]. Data showed that licorice could increase the expression of antigen presentation genes and promote CD8^+^ T cell infiltration within the circumstance of cell cycle arrest. So, we concluded that licorice induces G1 cell-cycle block in lung cancer cells by inhibiting CDK4-Cyclin D1 complex, which in turn increase antigen presentation and results in intra-tumoral CD8^+^ T cell infiltration. Consistent with the results of multiple studies, cell cycle blockade can activate anti-tumor immunity by increasing the immunogenicity of tumor cells [56] and can also increase the expression of PD-L1 to inhibit anti-tumor immunity [57]. Theoretically, licorice increases the infiltration of CD8^+ ^T cells into the TME, which may enhance the anti-tumor effect of anti-PD-L1. However, the expected enhancement effect was not observed in the combination of licorice and anti-PD-L1. We speculate that licorice may affect the activation of CD8^+^ T cells through the direct PD-1/PD-L1 signaling pathway of  T cells, which is consistent with the function of anti-PD-L1. Therefore, the combination of licorice and anti-PD-L1 did not show a synergistic anti-cancer effect. In fact, licorice has been known to promote maturation and differentiation of lymphocyte in order to activate the immune system [58, 59]. In the subsequent research, we will focus in-depth research and verification on this problem.

In summary, this study evidenced that licorice induced G0/G1 phase cell cycle arrest by down-regulating CDK4-Cyclin D1 complex on tumor cells. In addition, licorice increased the expression of antigen presentation genes and infiltration of CD8^+^ T cells in TME . Therefore, this study illuminated a novel mechanism of anti-tumor effect of licorice in NSCLC treatment, and provide functional evidence for the development of natural products in anti-tumor immunity.

## Methods

### Pharmacokinetic evaluation

The ingredients of licorice were identified based on Traditional Chinese Medicine Systems Pharmacology Database (TCMSP, http://tcmspw.com/) [60] and Bioinformatics Analysis Tool for Molecular mechANism of Traditional Chinese Medicine (http://bionet.ncpsb.org.cn/batman-tcm/), [61], and active ingredients (shown in Table 1) were further screened out by the in silico ADME system, with the criteria of oral bioavailability (OB) ≥ 50%, drug-likeness (DL) ≥ 0.40.

### Target fishing and validation

We identified direct and indirect targets of licorice on the basis of two *in-house* computational methods: WES and SysDT. The WES model was introduced to detect drug direct targets of the active ingredients based on a large-scale of 98,327 drug-target relationships. As a novel tool, the obtained model performs well in predicting the binding with average sensitivity of 85% (SEN) and the non-binding patterns with 71% (SPE) with the average areas under the receiver operating curves (ROC, AUC) of 85.2% and an average concordance of 77.5% [62]. SysDT is performed with the combination of the chemical, genomic and pharmacological information based on Random Forest (RF) and Support Vector Machine (SVM) for target identification effectively. The obtained model is served as a valuable platform for prediction of drug-target interactions with an overall accuracy of 97.3%, an activated prediction accuracy of 87.7% and an inhibited prediction accuracy of 99.8% [63].

Then obtained targets were uploaded to Uniprot (http://www.uniprot.org) [64] to normalize their name and organisms. And the targets of Homo sapiens were chosen for further investigation. We used Cytoscape 3.7.0 software to construct and analyze compound-target network.

### GO enrichment analysis and KEGG analysis for targets

GOenrichment analysis and KEGG analysis were performed through mapping targets to DAVID (http://david.abcc.ncifcrf.gov) for classification. We chose the terms with *P* value less than 0.05.

### Cell proliferation assay

Cellular proliferation was assayed using a Cell Counting Kit‐8 (CCK‐8, Beyotime, China). In brief, 1 × 10^4 ^cells were seeded in 96‐well microplates. After 24 h, cells were treated with different concentrations of licorice or vehicle for 48 h. Then, 10μL CCK‐8 solution was added to each well and incubated at 37 °C for 4 h. Absorbance at 450 nm was measured using a microplate reader (Molecular Devices, California, USA).

### Cell lines, compounds, and reagents

H1975 A549 cells (National Collection of Authenticated Cell Cultures, Shanghai, China) were maintained in RPMI 1640 medium (C11875500BT, Gibco, Thermo Fisher Scientific) supplemented with 10% fetal bovine serum (10099141, Gibco, Thermo Fisher Scientific).

Licorice powder was purchased from LEMETIAN MEDICINE. And Typical HPLC chromatogram of licorice extract performed by LEMETIAN MEDICINE (Additional file 1: Figure S1).

### FACS analysis of cell cycle

Once H1975 cells achieved a 70% to 80% confluency, they were treated with 0.1% DMSO or different concentration of licorice for 48 h. Then, cells were fixed with ice-cold 70% ethanol at − 20 °C overnight. After fixation, cells were washed thrice with cold PBS and then stained with Cell Cycle and Apoptosis Analysis Kit (C1052, Beyotime Biotechnology) according to the manufacturer’s instructions. Samples were then analyzed using a NovoCyte Flow Cytometer (ACEA Biosciences). The results were analyzed by Flow Jo software (BD bioscience).

### Western blotting

For western blot analysis, cells or tumor tissue were lysed in lysis buffer from the Qproteome Mammalian Protein Prep Kit (37901, QIAGEN) with the addition of protease inhibitors after PBS washing. Protein concentrations were measured by a microplate reader (Molecular Devices, California, USA) using the BCA Protein Assay Kit (P0010S, Beyotime, China). Then equal amounts of protein were resolved on SDS-PAGE and transferred to nitrocellulose membranes (Millipore, Bedford, MA, USA) and incubated with primary antibodies against: CDK4 (1:5000, ab108357, Abcam), cyclin D1 (1:1000, 554180, BD Bioscience, USA), cyclin  A2 (1:2000, ab181591, Abcam), cyclin B1 (1:50000, ab32053, Abcam), P21 (1:5000, ab109520, Abcam), PD-L1 (1:500, ab205921, Abcam or 1:2000, PA5-28115, Thermo Fisher scientific) and β-actin (1:2000, ab8227, Abcam); Secondary antibodies were goat anti-rabbit HRP (1:10000, ab6721, Abcam) and goat anti-mouse HRP (1:5000, ab97023, Abcam). Immunoreactive polypeptides were detected by electrochemiluminescence (ECL) reagents (Cat#170-5061, Bio Rad) using ChemiDoc™ XRS + Imaging System (Bio-Rad). Western blot band intensity quantification was calculated using ImageJ.

### Cell synchronization and FACS analyses

For synchronization into the G2/M phase of the cell cycle progression, H1975 cells were treated with 100 ng/mL of nocodazole (M1404, Sigma-Aldrich) for 16 h. Then cells release was collected at the indicated time points and fixed by 70% ethanol at − 20 °C overnight. After fixation, cells were washed 3 times with cold PBS and stained with Cell Cycle and Apoptosis Analysis Kit (C1052, Beyotime Biotechnology) according to the manufacturer’s instructions. Stained cells were sorted with NovoCyte Flow Cytometer (ACEA Biosciences). The results were analyzed by Flow Jo software (BD bioscience).

### Experimental model in vivo and subject details

All animal protocols described in this study were approved by the Institutional Animal Care and Use Committee (IACUC: 2018120202) at The Kanion Parmaceutical. C57BL/6 female mice (purchased from The Comparative medicine center of Yangzhou University) with 6–8 weeks of age were used. To generate tumor model, 5 × 10^5^ LLC cells/mouse were injected into the flanks of mice. Licorice (200 mg/kg of body weight) was administered daily by gastric gavage from day 2 after inoculation; Anti-PD-L1 (B7-H1) (10F.9G2) (BE0101, BioXCell) was administered by intraperitoneal (i.p.) injection on day 4, 7, and 10 after inoculation (200 ug of each mouse); control mice were treated with vehicle (0.9% NaCL) 5 ml/kg by i.p. injection. Tumor volume was measured once every two days when diameter of tumor reached 5 × 5 mm, and tumor volume was calculated by using the formula: 1/2 × length × width^2^. Mice with tumors greater than 2000 mm^3^ were sacrificed and tumors were collected and snap-frozen. And mice body weight of all the groups was also recorded every three days during the experiment.

### Real-time RT-PCR analyses

Total RNAs were extracted using the RNeasy mini kit (74106, QIAGEN), and reverse transcription reactions were performed using the Prime Script RT reagent Kit with gDNA Eraser (Perfect Real Time) (RR047A, Takara). After mixing the generated cDNA templates with primers/probes and Green® Premix Ex Taq™ II (Tli RNaseH Plus) (RR820B (A × 2), Takara), reactions were performed with the Step One Plus TM Real-Time PCR System (Applied Biosystems).

Mouse GAPDH: Forward, 5′-AGGTCGGTGTGAACGGATTTG-3′,

Reverse, 5′-GGGGTCGTTGATGGCAACA-3′;

Mouse Tap1: Forward, GGACTTGCCTTGTTCCGAGAG,

Reverse, GCTGCCACATAACTGATAGCGA;

Mouse Tap-2: Forward, CTGGCGGACATGGCTTTACTT,

Reverse, CTCCCACTTTTAGCAGTCCCC;

Mouse Tap-bp: Forward, GGCCTGTCTAAGAAACCTGCC.

Reverse, CCACCTTGAAGTATAGCTTTGGG.

Mouse Erap1: Forward, TAATGGAGACTCATTCCCTTGGA.

Reverse, AAAGTCAGAGTGCTGAGGTTTG.

### Single cell generation from tumor tissue and flow cytometry analysis

Tumor tissues were minced and digested with Collagenase IV (2 mg/ml, 17104-019, Gibco) and DNase I (2000U/ml, D7073, Beyotime) and Hyaluronidase (0.5 mg/ml, S10060, YuanYe Biotechnology) and Dispase II (0.5 mg/ml, S25046, YuanYe Biotechnology) in DMEM for 30 min at 37 °C. Cells were then collected by centrifuge and filtered through a 70 μm strainer (15–1070 BIOLOGIX) in DMEM. Cell pellets were suspended and lysed in red blood cell lysis buffer (Beyotime Biotechnology) for 5 min. The cells were then filtered through a 40 μm strainer (15-1040, BIOLOGIX) in 1 × PBS with 2% BSA. 1×10^6^ cells were incubated with antibodies against Anti-mouse CD3e APC (145-2C11) (05122-80-25, Biogems), Anti-mouse CD8a PE (53-6.7) (100707, BioLegend), Anti-mouse CD45 PE/Cy7 (30-F11) (103114, BioLegend) at room temperature for 30 min. Cells were washed by 1 × PBS with 2% BSA 3 times and detected by NovoCyte Flow Cytometer (ACEA Biosciences).

### Elisa analysis

Cytokines of mouse serum in licorice-treated group and control group were analyzed according to the manufacturer’s recommendations: Mouse IFN-γ Immunoassay (Cat#MIF00, R&D, USA.). Absorbance was measured on a microplate reader (Molecular Devices, California, USA) using Prism 8.0.2 (GraphPad Software, Inc.).

### Quantification and statistical analysis

Statistical analyses were performed with Prism 8.0.2 (GraphPad Software, Inc.). Two groups comparison using student’s t test. Multiple comparisons using one-way analysis of variance (ANOVA) followed by Tukey test. Tumor volume were analyzed using two-way ANOVA followed by Tukey test. Differences were considered statistically significant at a p value ≤ 0.05. Data are presented as the mean ± SD. *p < 0.05, **p < 0.01, ***p < 0.001, ****p < 0.0001. All data shown is representative two or more independent experiments, unless indicated otherwise.

## Supplementary Information

**Additional file 1.** Table S1 Candidate targets for each active compound. Fig. S1 Additional file 1  Inhibition of Liquiritin on H1975 cells. Fig. S2 Typical HPLC chromatogram of licorice extract where: (1) Liquiritin, 2.28% (2) Liquiritigenin, 0.18% (3) Glycyrrhizin, 2.95% (4) Isoliquiritigenin, 0.035%.  Fig. S3 Regulation of the CDK4-Cyclin D1/PD-L1 axis with GUF in A549 cells. Fig. S4 Correlation between CD8^+^ T cell infiltration and licorice targets in TCGA LUAD dataset and infiltration of CD8^+^ T cells caused by GUF in LLC mouse model.

## Data Availability

All other data are included within the Article or Supplementary Information or available from the authors on request.
