# Supplementary material for: Licorice extract inhibits growth of non-small cell lung cancer by down-regulating CDK4-Cyclin D1 complex and increasing CD8+ T cell infiltration
Source: Cancer Cell Int. 2021 Oct 12;21:529. doi: 10.1186/s12935-021-02223-0 (PMC8507331; doi:10.1186/s12935-021-02223-0)
Supplement: Supplementary file 1 — Additional file 1. Table S1 Candidate targets for each active compound. Fig. S1 Additional file 1 Inhibition of Liquiritin on H1975 cells. Fig. S2 Typical HPLC chromatogram of licorice extract where: (1) Liquiritin, 2.28% (2) Liquiritigenin, 0.18% (3) Glycyrrhizin, 2.95% (4) Isoliquiritigenin, 0.035%. Fig. S3 Regulation of the CDK4-Cyclin D1/PD-L1 axis with GUF in A549 cells. Fig. S4 Correlation between CD8+ T cell infiltration and licorice targets in TCGA LUAD dataset and infiltration of CD8+ T cells caused by GUF in LLC mouse model. [file 12935_2021_2223_MOESM1_ESM.docx]

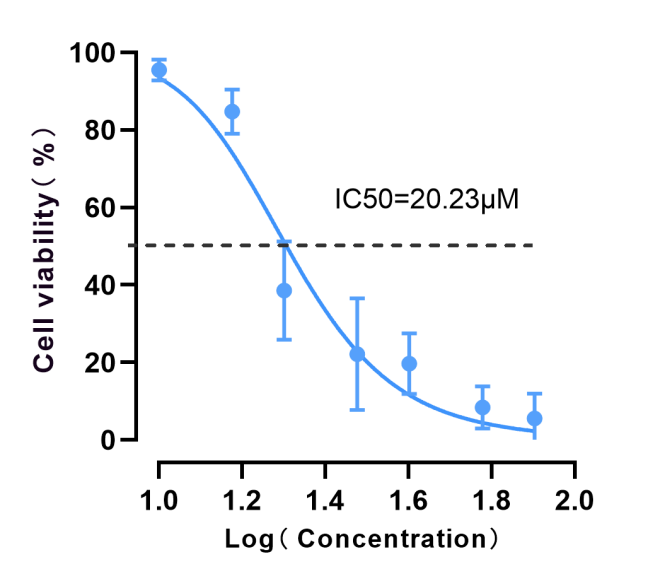


**Figure S1** Inhibition of Liquiritin on H1975 cells. H1975 cells were treated with different concentrations of Liquiritin (10, 15, 20, 30, 40, 60, 80μM) or for 48 h, cell viability was determined using the CCK-8 assay. Liquiritin inhibited H1975 cells growth significantly after 48 h treatments with IC50=20.23μM. (mean ± SD, n=6).


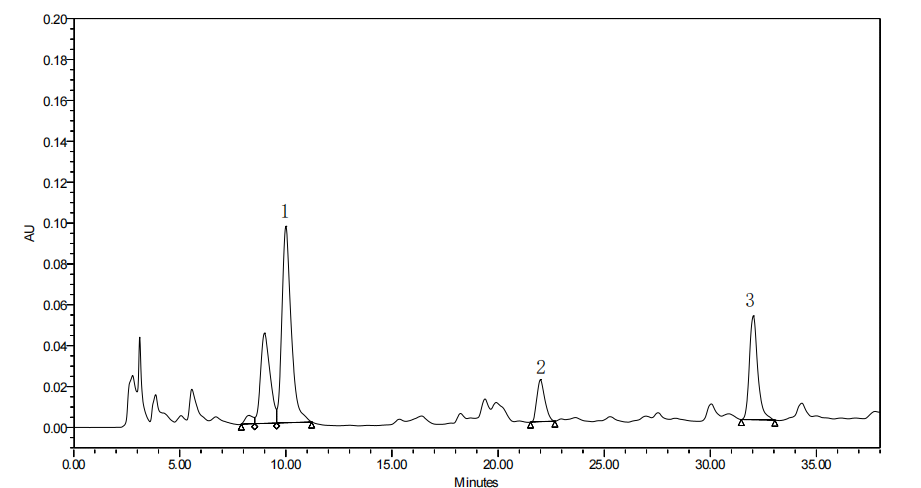

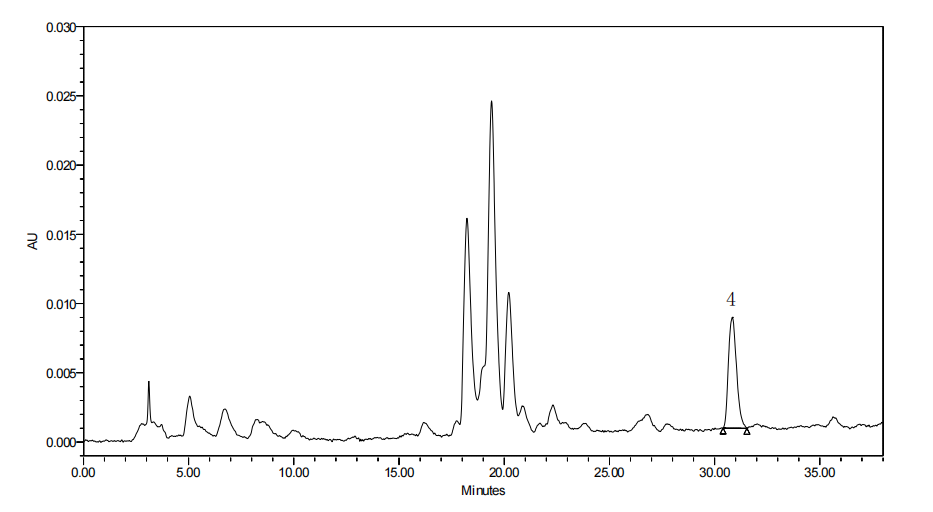


**Figure** **S2** Typical HPLC chromatogram of licorice extract where: (1) Liquiritin, 2.28% (2) Liquiritigenin, 0.18% (3) Glycyrrhizin, 2.95% (4) Isoliquiritigenin, 0.035%


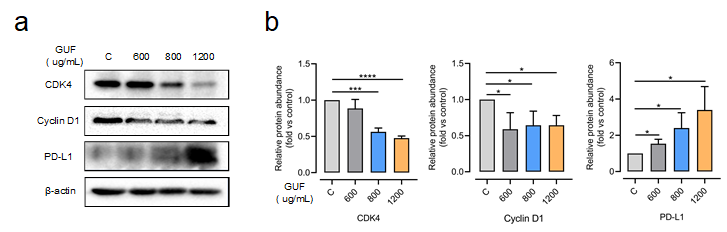


**Figure S3** Regulation of the CDK4-Cyclin D1/PD-L1 axis with GUF in A549 cells.

b The protein expression in A549 cells pretreated with 400, 600, and 800 µg/ml GUF or vehicle control for 48h, was measured by immunoblots, versus β-actin as a loading control (each band with three replicates). c Relative protein abundance of, CDK4, Cyclin D1, PD-L1 of (b), each bar represents mean ± SD from three replicates. (*p < 0.05, ***p < 0.001，****p < 0.0001.)


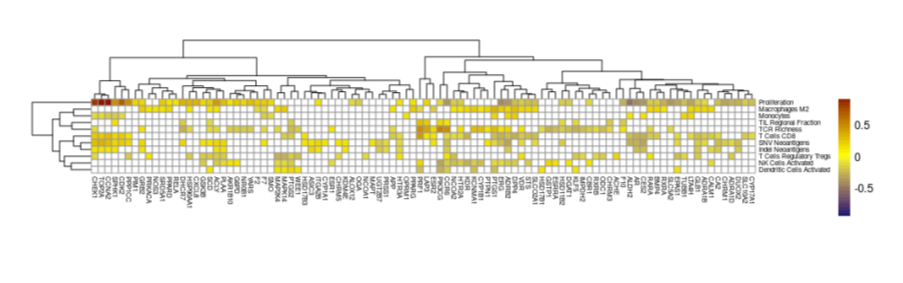

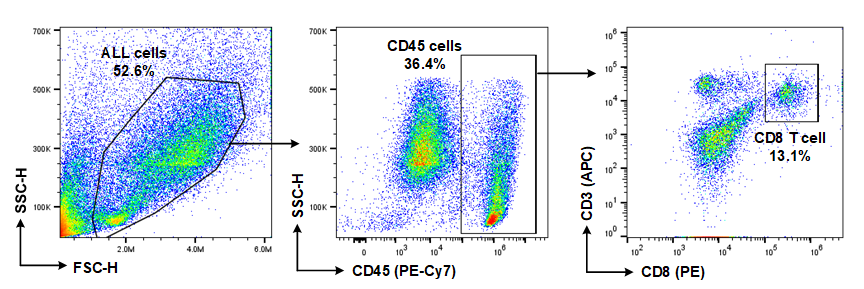

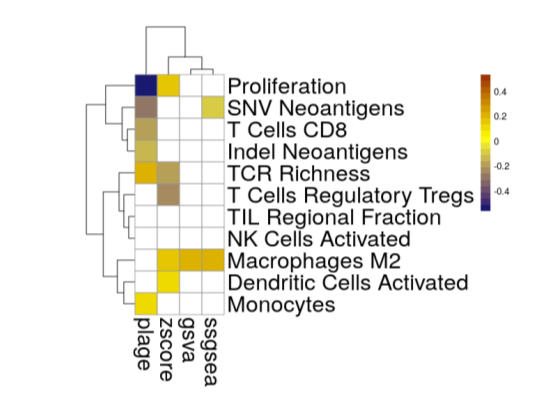

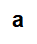

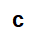

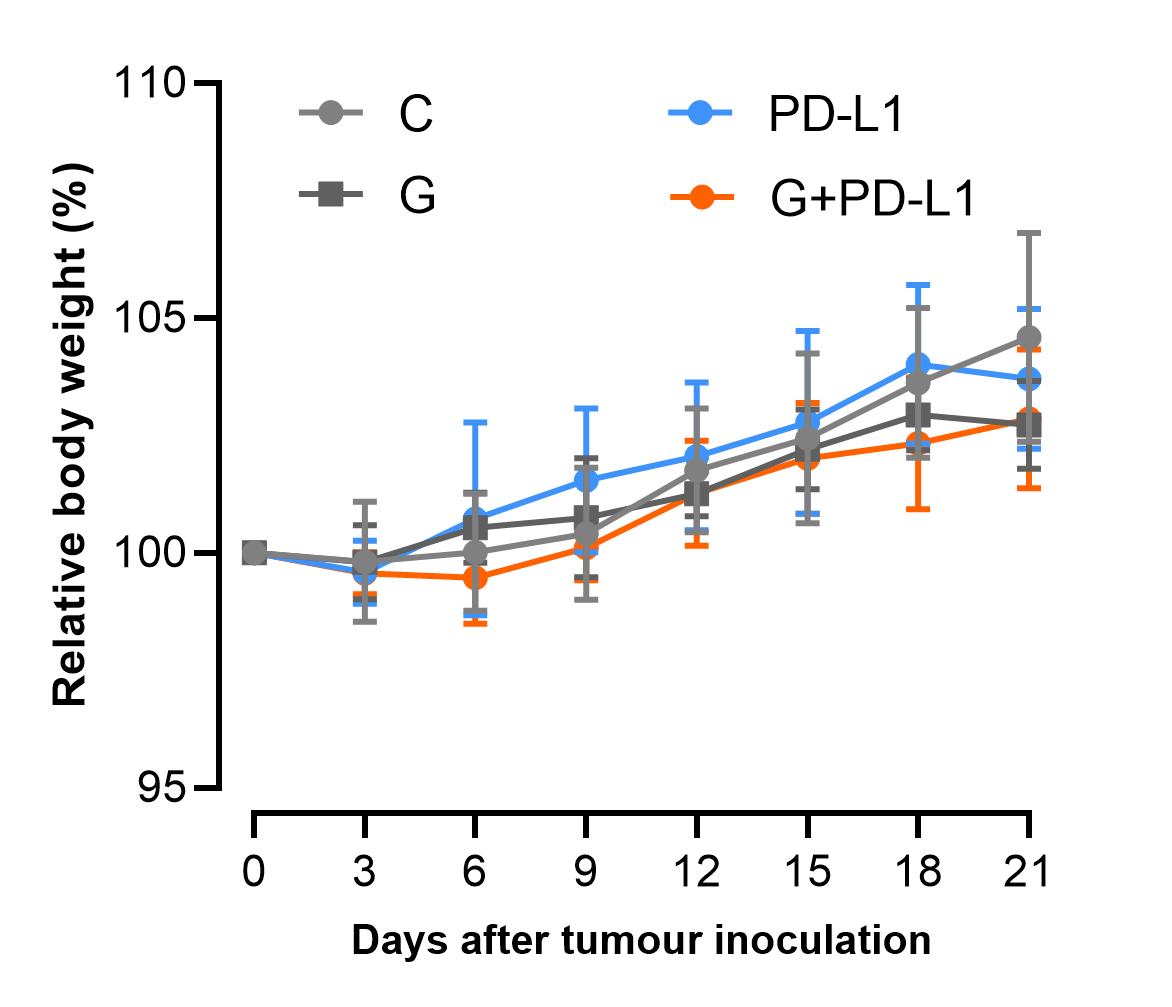

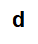

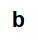

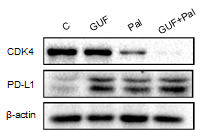

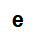


**Figure S4** Correlation between CD8^+^ T cell infiltration and licorice targets in TCGA LUAD dataset and infiltration of CD8^+^ T cell caused by GUF in LLC mouse model. a Relative change in body weight of mice treated with 200 mg/kg GUF or vehicle once daily from day 2 and/or 200 μg/mouse anti-PD-L1 (i.p.) on day 4, 7, 10 (n=5 per group). b Heatmap of Pearson’s correlation coefficients (PCCs) between gene expression level of licorice targets and immune phenotypes in TCGA LUAD dataset. The overall expression level of licorice targets was evaluated by four gene set analysis approaches (Single sample Gene Set Enrichment analysis (ssGSEA), Gene Set Variation analysis (GSVA), z-score, and Pathway Level Analysis of Gene Expression (plage)). *P* value≤0.05. c Heatmap of Pearson’s correlation coefficients (PCCs) between gene expression level of targets of licorice and immune phenotypes in TCGA LUAD dataset. the x-axis represents the targets of licorice. *P* value≤0.05. d Gating strategy for CD8^+^ T cell among cells of GUF-treated tumor tissue, T cells were assessed as CD45^+^; CD8^+^ T cells were assessed as CD3^+^CD8^+^, plots are representative of two experimental replicates. Numbers represent % cells within depicted gate. e Protein expression in H1975 cells treated with the 20nM Palbociclib and/or GUF was measured by immunoblots 48h later, versus β-actin as a loading control. Palbociclib has the same effect as GUF in increasing PD-L1 expression, and the combination has no obvious superimposed effect.

**Table S1. Candidate targets for each active compound.**

| **NO.** | **MOL-ID** | **Compounds** | **Potential targets** |
| --- | --- | --- | --- |
| M-158 | MOL001484 | Inermine | CALM1, ADRA1B, IGHG1, CA2, PTPN1, HTR3A, CHRM3, PTGS1, SCN5A, PRSS1, PRKACA, CHEK1, PIM1, NOS2, PTGS2, DPP4, MAPK14, PIK3CG, AR, GSK3B, CDK2, ESR2, HSP90AA1, CCNA2, KDM4E, SCD, ADCY5, STS, ESRRA, BMP4, CBR1, HTR2A, NR0B1, EPAS1, PLAA |
| M-226 | MOL000211 | Mairin | AKR1B10 , CYP17A1, RARA, G6PD, VDR, UGT2B7, SRD5A1, RXRA, PPP1CC, HSD11B2, ADCY1, SLC10A2, DUOX2, DHCR7, ESR1, AR |
| M-142 | MOL002311 | Glycyrol | F2, PIM1, PTGS2, NOS2, PPARG, MAPK14, GSK3B, CCNA2, ESR1, CHEK1, ADCY5, ESRRA, CBR1, WEE1 |
| M-131 | MOL004808 | glyasperin B | CES2, SCD, APP, LTA4H, MAPT, ESRRA, HTR2A, RELA, ACHE, NCOA2, CA2, CALM1, DPP4, TOP2A, KDR, F10, PIM1, F2, NOS2, PTGS2, F7, PRSS1, PTPN1, AR, CHEK1, GSK3B, PPARG, CCNA2, ESR2, CDK2, HSP90AA1 |
| M-134 | MOL004810 | glyasperin F | CALM1, F2, F10, DPP4, CA2, PRSS1, GSK3B, PTGS1, TOP2A, SCN5A, NOS2, PIM1, AR, PTGS2, PTPN1, CHEK1, PPARG, MAPK14, CCNA2, ESR2, ESR1, CDK2, HSP90AA1, CES2, ADCY5, STS, LTA4H, ODC1, HSD17B1, CBR1, ALOX12, HTR2A, ASIC3 |
| M-184 | MOL004820 | kanzonols W | SCD, APP, ALDH2, MAPT, ESRRA, ODC1, HSD17B1, ALOX12, HTR2A, HSD17B3, NR0B1, RELA, KLF5, ASIC3, NCOA2, TOP2A, SCN5A, F10, ACHE, F2, PTGS1, CALM1, CA2, NCOA1, NOS2, PTGS2, CHEK1, GSK3B, AR, PTPN1, PRSS1, PIM1, RXRA, MAPK14, PPARG, CCNA2, ESR2, CDK2 |
| M-219 | MOL004855 | Licoricone | CA2, NCOA2, CALM1, TOP2A, PRSS1, F10, PIM1, AR, KCNH2, F2, PTGS2, NOS2, PTPN1, KDR, CHEK1, PPARG, CES2, STS, ESRRA, ODC1, CBR1, HTR2A, TUBB1 |
| M-48 | MOL004863 | 3-(3,4-dihydroxyphenyl)-5,7-dihydroxy-8-(3-methylbut-2-enyl)chromone | NCOA2, CALM1, F10, F2, NOS2, PIM1, AR, PRSS1, PTPN1, CHEK1, PTGS2, PPARG, GSK3B, CCNA2, MAPK14, CDK2, HSP90AA1, ADCY5, CYP1A1, LTA4H, ESRRA, ACHE, HTR2A, RELA, ASIC3, PLAA |
| M-141 | MOL004879 | Glycyrin | NCOA2, CA2, CALM1, TOP2A, DPP4, F10, PRSS1, KCNH2, PIM1, AR F2, KDR, PTGS2, NOS2, CHEK1, ESR2, PPARG, ESRRA, IMPDH2, HTR2A, RELA, TUBB1 |
| M-198 | MOL004885 | licoisoflavanone | PTGS1, F2, ACHE, F10, CALM1, CA2, SCN5A, F7, TOP2A, NOS2, NCOA1, PTGS2, GSK3B, PIM1, AR, PRSS1, CHEK1, PTPN1, PPARG, CCNA2, ESR1, ESR2, CDK2, HSP90AA1, CES2, STS, ODC1, ALOX12, HTR2A, ASIC3, PLAA |
| M-262 | MOL004891 | shinpterocarpin | ADCY5, ALDH2, STS, ERG, ODC1, HSD17B1, HTR2A, MAP2K4, ASIC3, CA2, F2, ADRA1B, CALM1, ADRA1D, CHRNA7, NCOA1, PTGS1, ACHE, PRSS1, DPP4, PRKACA, RXRB, CHRM3, PTPN1, SCN5A, CHEK1, PIK3CG, PTGS2, PIM1, GSK3B, MAPK14, NOS2, CHRM5, CHRM1, PPARG, ADRB2, OPRM1, RXRA, AR, CDK2, CCNA2, OPRD1, ESR2, ESR1 |
| M-223 | MOL004903 | liquiritin | CA2, AR, CALM1, F7, F10, NOS2, CCNA2, F2, PPARG, DPP4, ESR1, OGA, ITGA2B, STS, LTA4H, GLB1, HTR2A, LAP3, SLC5A2, ACLY, YARS |
| M-202 | MOL004904 | licopyranocoumarin | SCD, APP, STS, LTA4H, MAPT, HTR2A, RELA, ASIC3, CA2, ACHE, CALM1, TOP2A, F10, F2, PTGS2, KDR, F7, PRSS1,, PIM1, AR, NOS2, PTPN1, CCNA2, PPARG, CDK2 |
| M-123 | MOL004908 | Glabridin | DPP4, NCOA2, F2, ADRA1B, IGHG1, CALM1, CA2, ACHE, PRKACA, CHRM1, SCN5A, NOS2, GSK3B, NCOA1, CHEK1, PTGS2, PTPN1, PRSS1, PIM1, MAPK14, RXRB, ADRB2, AR, RXRA, PPARG, CCNA2, CDK2, ESR2, SCD, APP, STS, LTA4H, MAPT, ESRRA, ODC1, HSD17B1, HTR2A, NR0B1, ASIC3 |
| M-126 | MOL004912 | Glabrone | CES2, SCD, DGAT1, APP, ALDH2, STS, LTA4H, MAPT, ODC1, ALOX12, HTR2A, HSD17B3, NR0B1, EPAS1, ASIC3, DPP4, ACHE, F10, CALM1, PTGS1, F2, CA2, SCN5A, NOS2, PTGS2, PRSS1, AR, GSK3B, PIM1, PTPN1, RXRA, CHEK1, MAPK14, PPARG, CCNA2, ESR2, ESR1, CDK2 |
| M-22 | MOL004914 | 1,3-dihydroxy-8,9-dimethoxy-6-benzofurano[3,2-c]chromenone | AR, MAPK14, GSK3B, PTPN1, PRKACA, PPARG, ESR1, CHEK1, CDK2, HSP90AA1, CCNA2, ESR2, ADCY5, CYP1A1, PRF1, CBR1, WEE1, CCR6, NR0B1, TUBB1 |
| M-29 | MOL004959 | 1-Methoxyphaseollidin | CYP1A1, CYP1A2, CYP1B1, SLCO2A1, NOS3, NCOA2, ADRA1D, CALM1, TOP2A, F10, ADRA1B, PRSS1, NCOA1, KDR, KCNH2, F2, PIM1, DPP4, PTGS1, PPARG, NOS2, GSK3B, AR, RXRA, MAPK14, CHEK1, PTGS2, ADRB2, SCN5A, CDK2, PIK3CG, ESR2, CCNA2, ESR1, HSP90AA1 |
| M-112 | MOL005001 | Gancaonin H | NCOA2, CA2, CALM1, PTGS2, PIM1, AR, TOP2A, KDR, F10, CCNA2, PRSS1, PTPN1, ESR1, HSP90AA1, SCD, DGAT1, CYP3A4, F7, GSTP1, LTA4H, MAPT, ESRRA, ODC1, HSD17B1, ALOX12, HTR2A, RELA, SMO, SPHK1, ASIC3 |
| M-187 | MOL005003 | Licoagrocarpin | SCD, APP, STS, LTA4H, MAPT, ESRRA, CBR1, HTR2A, MTNR1A, TUBB1, NCOA2, NOS3, CA2, CALM1, F10, ACHE, ADRA1B, DPP4, CHRM3, F2, PRSS1, PIM1, KCNH2, CHEK1, PTGS2, MAPK14, NOS2, RXRA, GSK3B, SCN5A, CDK2, AR, PPARG, ADRB2, CCNA2, ESR2, HSP90AA1 |
| M-137 | MOL005007 | Glyasperins M | NCOA2, KCNMA1, PTGS1, DPP4, CA2, CALM1, F2, ACHE, F10, TOP2A, KDR, PTGS2, KCNH2, PRKACA, F7, PIM1, GSK3B, NOS2, NCOA1, PPARD, PRSS1, CHEK1, PTPN1, SCN5A, AR, PPARG, CCNA2, ESR1, CDK2, ESR2, HSP90AA1, CES2, STS, LTA4H, CXCL8, ODC1, HTR2A, TUBB1, ASIC3 |
| M-188 | MOL005012 | Licoagroisoflavone | CES2, SCD, DGAT1, CYP1A1, APP, ALDH2, STS, GSTP1, MAPT, ESRRA, ALOX12, HTR2A, NR0B1, KLF5, EPAS1, ASIC3, F10, CA2, CALM1, SCN5A, F2, DPP4, NOS2, PIM1, PTGS2, PTPN1, AR, PRSS1, CHEK1, ESR2, MAPK14, GSK3B, PPARG, CCNA2, ESR1, CDK2 |
| M-250 | MOL005017 | Phaseol | AR, KDR, F2, NOS2, PIM1, PTPN1, PTGS2, MAPK14, PPARG, GSK3B, PRKACA, CDK2, CCNA2, HSP90AA1, CHEK1, ESR2, ADCY5, CBR1, ALOX12, WEE1, CCR6 |
| M-145 | MOL005008 | Glycyrrhiza flavonol A | ACHE, TOP2A, CALM1, F10, DPP4, CA2, F7, PIM1, PTGS2, AR, PRSS1, NOS2, PTPN1, GSK3B, CCNA2, ESR2, HSP90AA1, ESR1, CDK2, SCD, STS, MAPT, ESRRA, ODC1, HTR2A, HSD17B3, NR0B1, GRB2, HSD11B2, ASIC3 |
